# The Yellow Oxide of Mercury in Ophthalmic Practice

**Published:** 1882-05

**Authors:** F. C. Hotz

**Affiliations:** Ophthalmic Surgeon to the Illinois Charitable Eye and Ear Infirmary, Chicago


CHICAGO MEDICAL

### JOURNAL & EXAMINER.

Vol. XLIV.—MAY, 1882.—No. 5.

### Original Communications.

### Article I.

### The Yellow Oxide of Mercury in Ophthalmic Practice.

By F. C. Hotz, m.d., Ophthalmic Surgeon to the Illinois Charitable Eye and Ear Infirmary, Chicago.

The yellow oxide of mercury (hydrargyrum oxyd. flavum) is an impalpable powder, which, when properly mixed with vaseline, makes an ointment of the greatest uniformity, and on this account is preferred by oculists to the red oxide. It is a powerful local stimulant and alterative, promoting the absorption of the products of inflammation, and encouraging the process of repair; but we know little about the exact way in which it accomplishes this effect, although it has been in use these twenty years.

It owes its introduction into ophthalmic therapy principally to the late Dr. H. Pagenstecher, of Germany, upon whose recommendation, in 1861, it was methodically employed in many eye-clinics.

But though it showed itself worthy of the high praise bestowed upon it by Pagenstecher, whenever it was tried, it seems to me it has not gained yet that wide and general recognition it merits. At home and abroad I have even quite recently met with cases which had been treated by various remedies unsuccessfully, where a few applications of "Pagenstecher's yellow salve" would achieve a speedy cure. One of the best instances of this kind I saw in an eye infirmary two years ago. A case of fascicular keratitis (for which I regard the yellow salve almost in the light of a specific) had been treated with eserine for three weeks without showing the slightest improvement; still eserine was continued, though the yellow salve usually subdues an inflammation in three or four days.

It probably appears as if I have a biased opinion of the remedy; but I hope I shall prove that my partiality, as far as it goes, is well grounded. But while I value the remedy very highly, I am far from following the example of a recent writer, who seems to regard the yellow oxide as a cure-all for almost every inflammation of the conjunctiva and cornea. In his publications he denounced the use of astringents for catarrhal conjunctivitis in terms which leave no doubt about the sincerity of his opinion; they should be abandoned, and yellow oxide be used in their stead. Without questioning the truth of his statement in regard to the happy results he obtained by his plan of treatment, I, for my part, have been so well satisfied with the good effect of mild astringents in the treatment of the simple forms of conjunctivitis, that I feel not disposed to abandon their use.

But there are three diseases of the eye for which I consider the yellow oxide preëminently superior to any other local remedy. These are: 1. One form of blepharitis ciliaris; 2. Phlyctenular conjunctivitis; 3. One form of phlyctenular keratitis.

1. Under the name, "blepharitis ciliaris" (or ophthalmia tarsi), the text-books describe two distinct affections which ought to be distinguished from each other by different names. Both affections have their seat in the sebaceous glands of the lid border, the one being due to inflammation, the other to hypersecretion of these follicles. The inflammation produces pustules and follicular ulcers around the eyelashes; the hypersecretion causes the production of epidermis-like scales. I have, therefore, distinguished the two affections in my records as blepharitis *ulcerativa* and *desquam-*

*ativa.* But we might as well adopt the nomenclature of the dermatologists, who distinguish the similar diseases of the skin as *acne* and *seborrhœa.*

Now, the form of blepharitis to which I wish to invite the attention of the reader for a few moments, is the seborrhœa or blepharitis desquamativa. Where the disease is well marked, you find along the rows of eyelashes epidermis-like scales of various size and thickness ; sometimes they accumulate layer upon layer to form small heaps around one or more eyelashes. These scales can be wiped off by the finger or a soft cloth, or scraped off by the finger-nail. The skin underneath may be reddened, but otherwise it is unchanged, showing neither pus spots nor ulceration. In the milder forms, however, the scales are not so conspicuous ; they are very fine and thin, and on this account, often escape the attention of the surgeon. And in some cases, where I suspected the lid border being the source of the patient's complaints, it appeared at first quite normal, but when a little yellow ointment was applied, numerous minute scales could be rubbed off, which before were invisible. In doubtful cases, therefore, I can recommend the use of the yellow ointment as a very good means of deciding the diagnosis.

Such eyelids are exceedingly sensitive to external influences; exposure to impure or cold air, winds, heat, gaslight, make them red, and the patient is annoyed by an unpleasant burning and itching sensation in his eyelids. But the principal complaint is a sort of asthenopia ; the patient comes to you saying he has got "weak eyes;" he cannot enjoy the free use of his eyes for reading, writing, or any other close application ; sooner or later he is obliged to abandon work because his eyes ache, and burn, aad weep, and feel as if they were full of smoke or dust ; sometimes ordinary daylight even is unbearable to such eyes.

The source of these subjective symptoms is not the seborrhœa itself as much as the hyperæmia of the tarsal conjunctiva, which is always associated with it. And since the conjunctival hyperæmia sufficiently accounts for all the subjective complaints, many physicians and oculists overlook the seborrhœa, or, if they notice it, regard it as secondary in nature and importance, and concentrate their whole therapeutic effort upon the conjunctival hyper-

æmia. The whole list of astringent eye-waters is tried successively; or the conjunctiva is touched occasionally with a crystal of alum or copper. Month after month such patients have been treated for "granular lids," when the most thorough examination failed to discover even a microscopic evidence of past or present granulations, but simply found hyperæmia of the conjunctiva dependent on blephar. cil. desquamativa. The result of this wrong treatment can easily be imagined. It hurts both the patient and the physician. It hurts the patient physically, because it aggravates his complaint; and it hurts the physician morally, when by another physician his patient is quickly relieved of a complaint which he had been fighting unsuccessfully for so many months.

Ignore the secondary conjunctival hyperæmia; direct your whole attention to the primary trouble; regulate the action of the tarsal glands, and you will speedily relieve your patient of all his complaints, because the conjunctival congestion will subside *pari passu* with the seborrhœa. Two instances may be related for the sake of illustration. A young lady, while attending a boarding-school abroad, last summer, experienced some asthenopic trouble; she could not read more than twenty minutes, when she was obliged to quit on account of severe burning and aching; her eyes became red and felt very uncomfortable during the rest of the day. A very competent oculist whom she consulted diagnosticated "granular eyelids," and treated the case accordingly. The treatment was very painful, and unsuccessful. Under these circumstances the lady decided to quit school and return home. When she consulted me, I found a slight bleph. cil. desquam., $V=\frac{20}{20}$; under homatropine H 1-50; simple hyperæmia of the conjunctiva of the upper lids, aud the conjunctiva of the lower lids very red and swollen. This swollen condition I attributed to the effect of the previous treatment, and took no further notice of it. Four applications of the yellow salve relieved the young lady of all her eye trouble, and she could read again all day without the least discomfort.

Another instance is the case of a lady, aged twenty-two years. Her eyes had been "weak" the past three years. Last year they had been treated with the alum crystal for six months without any benefit. At her first visit her eyes were very red, as if she had just been crying; the borders of the eyelids were thickly covered with sebaceous dandruff-like scales; the conjunctiva was intensely bright red, but thin and smooth, and there was no secretion of mucus. R. E. V=$\frac{20}{200}$ only; L. E. V=$\frac{20}{20}$, and Em. also under homatropine. The cleaning of the lids and the application of the yellow salve made them very red; but on the next day they appeared much better, and within four weeks they were cured.

But in order to secure these good results, I deem it necessary to give the case my personal attention, or to instruct some attendant or friend of the patient in all the minutest details of the treatment. If the patient is simply told to rub the ointment in his eyelids, he will probably spread it indiscriminately on any other part of the lid but the border which should be anointed. You must show him how and where to apply the ointment, and in the first place, you must impress on his mind the absolute necessity of cleaning the lid borders of all scaly matter prior to the inunction. Show him how easily and harmlessly these scales can be removed with the fingers or a soft cloth. When the eyelids are scrupulously clean, take with the end of a probe a small amount of the ointment (as much as the size of a pin's head is sufficient for one eyelid), and spread on the skin along the row of eyelashes. If the lower eyelid is to be treated, it is drawn down with the fingers of one hand, and the ointment spread below the eyelashes is thoroughly rubbed upward into the ciliary border with the finger of the disengaged hand. To treat the upper lid, it is best to close the eye, and by a slight traction upon the skin of the upper lid to evert its free border just enough to bring it in view; then the salve, spread out above the eyelashes, is rubbed down into them. When the application is made in this manner, it is not likely that any of the ointment will get beyond the row of eyelashes upon the posterior edge of the free border, whence it would easily find its way upon the conjunctiva, and cause unnecessary irritation and pain; but when any particles of ointment are found there, they should be wiped off with a soft cloth. The salve must be rubbed in so thoroughly that nothing but a faint greasy appearance of the ciliary edge betrays its presence. One such inunction each morning is usually sufficient to relieve even the worst degrees of bleph. cil. desquamativa.

2. The seat of the *phlyctenular conjunctivitis* is the ocular conjunctiva. Either at the sclero-corneal border or at a short distance from it, we find the epithelium lifted up by small grayish nodules, ranging in size between a millet and hemp seed. The development of these nodules or *phlyctenules* is attended by more or less hyperæmia of the ocular conjunctiva. When there is but one phlyctenule, the hyperæmia is often limited to a triangular section, having its basis in the retro-tarsal portion of the conjunctiva, and its apex at the seat of the efflorescence. When there are two or more phlyctenules, the vascular injection is proportionately more extended, so that in some cases the entire ocular conjunctiva is engorged, as if we had to deal with a very serious inflammation.

In every phase of this disease the yellow ointment is the most useful remedy. The best mode of its application is this: Suppose the left eye is to be treated; we charge the end of a silver probe with a small amount of ointment; sitting in front of the patient, and holding the probe lightly like a writing pen with the finger of our right hand, we rest this hand on the left temple of the patient; with the fingers of the left hand we separate the eyelids sufficiently to evert the lower lid, and introduce the charged end of the probe from the outer canthus until it has reached about the center of the lower cul-de-sac; now removing our left hand from the eyelids to let them be closed, we slowly withdraw the probe through the canthus. By this manœuvre the ointment is taken off from the probe, while it passes out between the eyelids, and remains within the conjunctival sac; and by gently rubbing the eyelids to and fro over the eyeball (massage) it is uniformly distributed over the surfaces of the ocular conjunctiva. A few applications of this kind allay the inflammation and procure a speedy absorption of the phlyctenules.

Some surgeons employ calomel dust in the local treatment of this affection. In the very mild forms it is perhaps as efficient as the yellow otntment, but I know of cases in which calomel has disappointed the surgeon, while the yellow ointment promptly achieved the desired improvement.

Sometimes the case presents a mixed character; that is to say, the development of the phlyctenules is attended by considerable swelling and catarrhal secretions of the conjunctiva of the eyelids. Under these circumstances our local treatment must be governed by the prevailing disease. If the catarrhal symptoms predominate, astringent lotions (zinc sulph. or alum) should be applied first, to be followed by the yellow ointment as soon as the catarrhal symptoms subside. If the phlyctenular manifestations prevail, begin the treatment with the yellow ointment, and conclude it with adstringentia. And sometimes I have found the alternating plan (one day the astringent and the next day the ointment) the best to answer our purpose.

Absolute purity of the drug, and a careful preparation of the ointment are, of course, two essential requirements for success.

3. The *fascicular keratitis* (or circumscribed vascular corneitis) is a sub-species of the phlyctenular keratitis, and can easily be recognized by its peculiar local manifestations. In the transparent cornea, at a variable distance from its margin, we notice a small gray or grayish-yellow phlyctenule, which is connected with the limbus conjunctivæ by a narrow leash of fine and parallel blood-vessels. The vessels of this bundle (*fasciculus*; hence the term "fascicular" keratitis) are subconjunctival arteries and veins extended over the limbus on to the cornea. The width of this vascular band varies from a very fine red streak to a broad triangle; but its vessels are always closely packed together, showing neither ramification nor tortuosity; and they always terminate at the phlyctenule.

At the beginning the phlyctenula, probably, is formed at or near the margin of the cornea, and as the disease progresses, it is gradually pushed further and further on toward the center of the cornea in front of the blood-vessels. For often we can observe the development of a new phlyctenula at one point of the corneal margin, while at another point in the same eye, one is far advanced already toward the center of the cornea. In the further progress of the disease the phlyctenular nodule loses its epithelium, and is transformed into a small superficial ulcer (vascular ulcer). The infiltration or ulcer may travel across the entire cornea, carrying its vascular band across the center, and thus seriously obscuring the sight. But it may stop at any point in its progress, remain stationary for an indefinite period, and sooner or later gradually fade away. Its vasicular tail then disappears also, leaving a faint gray streak behind, which, in older cases, remains as a permanent blemish of the eye.

During the progressive stage, and also while the disease is stationary, the eye shows all the signs of acute irritation (redness, lachrymation, pain and photophobia). Children afflicted with this eye-trouble shun daylight, and bury their faces in the pillow or crouch in the dark corner of the room. Now, when we consider that this fascicular keratitis is a disease peculiar to children of a poor, delicate constitution, either from strumous diathesis, or from bad food, or from chronic indigestion; when we consider that those children, to whom plenty of fresh air and sunshine are the best and most needed tonics, are deprived of these luxuries for weeks, or even months, by their sore eyes; when we consider the evil effects of prolonged confinement upon their physical and mental development—then we may fully appreciate the high value of a remedy which possesses the power to arrest the progress of the inflammation at any stage, to allay the symptoms of irritation in a few days, and to secure a speedy and complete recovery. So certain is the control of the yellow ointment over the fascic. keratitis, that we may well call it the remedy *par excellence* for this disease. It is far superior to the calomel.

In several text-books it is recommended not to employ the ointment until the signs of irritation have pretty well subsided. I cannot endorse this as good advice, nor can I find any sound reason for it. My experience is that nothing will subdue the irritation quicker than the yellow ointment. I have found its magical influence the more brilliant, the greater the intensity of the inflammation. One application was sometimes sufficient to soothe the most intense pain, to relieve the most violent photophobia, and to enable the poor child to enjoy the fresh air with open eyes which had been spasmodically and hermetically closed many weeks.

Have I pictured the merits of the yellow ointment in too vivid colors? Let the following observations, recorded during the year 1880, give the answer:

1. Geo. S., æt. nine years; vascular phlyctenula in left eye; has been treated with calomel dust the past four months. Begun treatment with yellow ointment May 13, 1880; cured May 31.

2. Anna B., æt four years; left eye, marginal phlyctenules; right eye, vascular phlyctenule reaching to center of cornea; intense photophobia and blepharospasm. Has not opened the right eye for three months. Daily application of yellow ointment from August 4 to 14; occasional treatment till September 10, when eye well.

3. Alice K., æt. one year six months; vascular phlyctenula in right eye, near center of cornea; duration two months. Yellow ointment from April 20 to May 10. Complete recovery.

4. Will. R., æt. thirty-two; May 23, 1880. Since last fall right eye inflamed, and treated with calomel. Vascular triangle occupying lower temporal section of cornea; apex near the center. Yellow ointment. June 12, all traces of it gone.

5. Louis M., æt. eleven; September 5, 1880. Inflamed eyes for two years. Broad vascular band in lower half of cornea of each eye, extending to center, where small ulcer. Photophobia; blepharospasms; much redness of eyeballs. Yellow ointment. September 7, great improvement after two applications; September 20, eyes well.

6. Miss S., æt. fifty-seven; July 12, 1880. Seven years ago violent iritis of both eyes. Right eye hopelessly blind; left eye saved by iridectomy. Repeated attacks of iritis since, which always subsided by the use of atropia. Two weeks ago, left eye became very red and painful, and was not relieved by atropia. Could not bear any light; every movement of eyeball painful; profuse secretion of tears; intense pericorneal injection, especially around lower border of cornea. In lower half of cornea a narrow, semilunar gray ulcer, connected with the corneal margin by a broad vascular band. Yellow ointment, and Dover's powder internally. July 13, no pain; less photophobia; less redness. July 17, no pericorneal redness; ulcer clean and healing. July 29, eye well.

7. Amos R. G., æt. fifty-six; October 7, 1880. Had granular lids twenty years ago. Eyes inflamed three weeks. In upper half of each cornea a semilunar ulcer with sharp, infiltrated borders; broad vascular band from upper margin to ulcer. Intense pericorneal injection; pain and photophobia. Yellow ointment. Discharged cured October 31.

8. Benjamin G. M., æt. thirty; November 6, 1880. Semilunar ulcer in upper portion of the cornea, with vascular band; great pain and much redness. Yellow ointment. Discharged cured November 20.

9. Eugene Gr., æt. three; right eye, several large phlyctenules in cornea, near lower margin; vascular band to each. Yellow ointment. Under treatment from January 23 to February 14, and cured.

10. February 6; Minnie Z., æt. four; right eye, vascular ulcer in lower nasal section of cornea six weeks; great photophobia. Yellow ointment. February 8, eye open; can bear light quite well.

11 Mary F., æt. eighteen; right eye, vascular ulcer in upper half of cornea; much irritation, four weeks. Yellow ointment. Under treatment from March 5 to April 1. Eye well.

12. Child, eleven months old; right eye inflamed two weeks; swelling of tarsal conjunctiva, and much redness of ocular conjunctiva. In lower half of cornea a yellow infiltration, connected with margin by a vascular band. Treated with yellow ointment from July 3 to 12, and cured.

13. Minna L., æt. two; inflamed eyes since March. Small ulcer in center of each cornea; fine streak of vessels from lower margin to each ulcer. Cured by yellow ointment; under treatment from July 5 to 24.

14. Frank L., æt. four; infiltration in cornea of right eye, with vascular band; much photophobia. Yellow ointment July 10 to 28.

15. Annie Tr., æt. three; phlyctenule with vascular band in lower portion of cornea, right eye. Treated with yellow ointment from October 5 to 20.

16. Clara M., æt. two; right eye inflamed three weeks; phlyctenule with vascular band in temporal portion of cornea. Three treatments with yellow ointment removed inflammation and vascular band.

